# miR-10b-5p expression in Huntington’s disease brain relates to age of onset and the extent of striatal involvement

**DOI:** 10.1186/s12920-015-0083-3

**Published:** 2015-03-01

**Authors:** Andrew G Hoss, Adam Labadorf, Jeanne C Latourelle, Vinay K Kartha, Tiffany C Hadzi, James F Gusella, Marcy E MacDonald, Jiang-Fan Chen, Schahram Akbarian, Zhiping Weng, Jean Paul Vonsattel, Richard H Myers

**Affiliations:** Department of Neurology, Boston University School of Medicine, Boston, MA USA; Graduate Program in Genetics and Genomics, Boston University School of Medicine, Boston, MA USA; Bioinformatics Program, Boston University, Boston, MA USA; Center for Human Genetic Research, Massachusetts General Hospital, Harvard Medical School, Boston, MA USA; Friedman Brain Institute, Department of Psychiatry, Mount Sinai School of Medicine, New York, NY USA; Program in Bioinformatics and Integrative Biology, and Department of Biochemistry and Molecular Pharmacology, University of Massachusetts Medical School, Worcester, MA USA; Department of Pathology and Cell Biology, Columbia University Medical Center and the New York Presbyterian Hospital, New York, NY USA; Genome Science Institute, Boston University School of Medicine, Boston, MA USA

**Keywords:** Huntington’s disease, Human, Prefrontal cortex, Striatum, miRNA-sequencing, microRNA, miRNA, RNA biology, Age at onset, Neuropathological involvement

## Abstract

**Background:**

MicroRNAs (miRNAs) are small non-coding RNAs that recognize sites of complementarity of target messenger RNAs, resulting in transcriptional regulation and translational repression of target genes. In Huntington’s disease (HD), a neurodegenerative disease caused by a trinucleotide repeat expansion, miRNA dyregulation has been reported, which may impact gene expression and modify the progression and severity of HD.

**Methods:**

We performed next-generation miRNA sequence analysis in prefrontal cortex (Brodmann Area 9) from 26 HD, 2 HD gene positive, and 36 control brains. Neuropathological information was available for all HD brains, including age at disease onset, CAG-repeat size, Vonsattel grade, and Hadzi-Vonsattel striatal and cortical scores, a continuous measure of the extent of neurodegeneration. Linear models were performed to examine the relationship of miRNA expression to these clinical features, and messenger RNA targets of associated miRNAs were tested for gene ontology term enrichment.

**Results:**

We identified 75 miRNAs differentially expressed in HD brain (FDR q-value <0.05). Among the HD brains, nine miRNAs were significantly associated with Vonsattel grade of neuropathological involvement and three of these, miR-10b-5p, miR-10b-3p, and miR-302a-3p, significantly related to the Hadzi-Vonsattel striatal score (a continuous measure of striatal involvement) after adjustment for CAG length. Five miRNAs (miR-10b-5p, miR-196a-5p, miR-196b-5p, miR-10b-3p, and miR-106a-5p) were identified as having a significant relationship to CAG length-adjusted age of onset including miR-10b-5p, the mostly strongly over-expressed miRNA in HD cases. Although prefrontal cortex was the source of tissue profiled in these studies, the relationship of miR-10b-5p expression to striatal involvement in the disease was independent of cortical involvement. Correlation of miRNAs to the clinical features clustered by direction of effect and the gene targets of the observed miRNAs showed association to processes relating to nervous system development and transcriptional regulation.

**Conclusions:**

These results demonstrate that miRNA expression in cortical BA9 provides insight into striatal involvement and support a role for these miRNAs, particularly miR-10b-5p, in HD pathogenicity. The miRNAs identified in our studies of postmortem brain tissue may be detectable in peripheral fluids and thus warrant consideration as accessible biomarkers for disease stage, rate of progression, and other important clinical characteristics of HD.

**Electronic supplementary material:**

The online version of this article (doi:10.1186/s12920-015-0083-3) contains supplementary material, which is available to authorized users.

## Background

Huntington’s disease (HD) is an inherited disorder caused by a CAG trinucleotide repeat expansion in *HTT* which leads to progressive motor and cognitive impairment due to the gradual loss of neurons within striatal and cortical brain regions [1]. Although monogenic, HD displays remarkable variation in clinical expression, most readily observed by the range in age at clinical onset as determined by the manifestation of motor symptoms, varying from age 4 years to age 80 [2]. While onset age is unequivocally related to the size of the expanded CAG repeat, with longer repeats leading to earlier onset, only 50% to 70% of the variation can be attributed to repeat size [3,4]. The remaining variation is highly heritable (*h*^*2*^ = 0.56), suggesting a strong role for genes that modify disease progression [3].

MicroRNAs (miRNAs) are small non-coding RNAs that negatively regulate the expression of genes in a sequence-specific manner, binding to the 3′-untranslated region (3′UTR) to initiate cleavage or translational repression of target transcripts [5,6]. miRNAs influence a diverse range of cellular processes [7] and consequently, their altered expression may lead to or influence disease-related pathological phenotypes, or reveal unknown aspects of the disease process. In the central nervous system (CNS), miRNAs are abundant, as brain-specific miRNAs assist in various neuronal processes such as synaptic development, maturation and plasticity [8,9]. Altered miRNA expression has been observed in diseases of the CNS, particularly in age-dependent neurodegenerative diseases, which suggests that the expression of miRNAs may contribute to neuropathogenesis [10,11].

In HD, the dysregulation of miRNAs has been reported in HD *in vitro* models, transgenic HD animals and human HD brain [12-24]. We hypothesize that post-transcriptional regulation by miRNAs plays a role in modifying the progression and severity of HD. Recently, we completed a study of miRNA expression obtained through next-generation sequencing technology in human HD and control brain samples to investigate the presence of altered miRNA expression in HD and its role in transcriptional dysregulation [13]. The original study provided sample power to detect large miRNA changes, but the sample size was not sufficient to detect more subtle changes in miRNA expression and did not represent a wide enough range of HD pathology to investigate relationships to clinical features of HD. Therefore, to follow-up on these findings, we have sequenced small RNAs in an additional 16 HD brains, two of which are gene positive asymptomatic Vonsattel grade 0 cases, and 27 control samples, for a combined study of 28 HD and 36 control samples. The increased sample size enables the detection of significantly altered miRNAs with lower levels of differential expression as well as more comprehensive characterization of the relationship of these miRNAs to relevant clinical features of the disease, including the age at motor onset of the disease, disease duration (the time between onset and death), age at death and extent of pathological involvement in the striatum and cerebral cortex. A deeper understanding of the global miRNA expression in HD may elucidate pathogenic mechanisms of disease progression in HD and suggest new therapeutic targets.

## Results

### Differential expression analysis highlights disrupted miRNA expression in HD brain

To evaluate the relationship of miRNA expression to salient clinical and pathological features of HD, we profiled miRNA expression using small RNA-sequencing of prefrontal cortex (Brodmann Area 9) of 26 symptomatic HD and 36 control samples (see Table 1, Additional file 1: Table S1 and Additional file 2: Table S2). Although the striatum is the most affected brain region in HD, differences in miRNA expression between HD and unaffected controls, independent of cellular composition, would be difficult to assess due to the extent of neuron loss and the increase of reactive astrocytosis in HD striatal tissue [25]. Therefore, prefrontal cortex, which exhibits hallmark characteristics of HD pathology [26], relates to striatal involvement (Pearson r = 0.44, p < 2e-16) [27], but experiences less extreme changes than the striatum [28,29], was used for sequencing. In addition, previous studies have found no difference in cell counts between HD and controls from similar BA9 brain samples [13,30].

**Table 1:** **Summary of the brain samples used for miRNA-sequence analysis**

**Variable**	**HD, grades 2 - 4**	**Asymptomatic grade 0**	**Control**
N	26	2	36
Age at death	59.5 ± 10.7	67.5 ± 26.1	68.6 ± 14.3
RNA integrity number	7.3 ± 0.9	7.7 ± 0.6	7.7 ± 0.7
Post mortem interval	15.7 ± 7.7	28.0 ± 7.9	14.4 ± 8.8
CAG repeat size	44.6 ± 2.9	42.0 ± 0	
Age of onset	44.5 ± 11.8		
Disease duration	15.0 ± 6.1		
Striatal score	2.70 ± 0.65		
Cortical score	1.25 ± 0.50		

The HD samples consisted of Grade 2 (n = 4), Grade 3 (n = 15), and Grade 4 (n = 7) brains as determined by Vonsattel grade, an assessment of striatal involvement classified as 0 through 4 in order of the severity of neuropathological involvement [25]. Sequenced samples were also among the 523 HD brains characterized by the recently established measure of pathological involvement termed the Hadzi-Vonsattel score (H-V score), which independently characterizes both striatal and cortical pathological involvement in each brain [28]. While Vonsattel grading and H-V striatal score are closely related, (Pearson r = 0.90, measured using 346 HD brains), H-V scores are a continuous metric and therefore more amenable to adjustment of covariates such as CAG repeat size in modeling of neuropathological involvement and independently assesses striatal and cortical involvement. H-V scores ranged from 0–4, where 0 indicates no detectable neuropathological involvement and 4 indicates severe neuropathological involvement. Samples from symptomatic individuals had striatal scores ranging 1.43–3.82 and cortical scores ranging from 0.40–2.36 (see Table 1, Additional file 2: Table S2). Additionally, two Grade 0 brains (both with CAG repeat expansions of 42 repeats) were small-RNA sequenced and analyzed separately from the 26 HD brains used in differential expression analysis. Grade 0 brains were neuropathologically normal and asymptomatic at the time of death (see Table 1).

**Figure 1:**
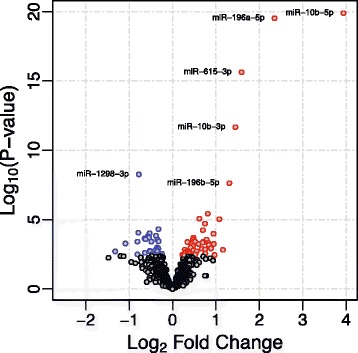
**Characterization of miRNA in Huntington’s disease brain.** Volcano plot of 75 significantly differentially expressed miRNA after FDR-adjustment for 938 comparisons. Points labeled red were up-regulated in HD and points labeled as blue were down-regulated in HD. Hox-related miRNA points are labeled and represent the top differentially expressed miRNA in HD.

After processing sequencing data to remove sequencing artifacts, normalize using variance stabilization transformation, and adjust for batch effects (see Methods), 938 miRNAs were reliably quantified and 75 of these were significantly differentially expressed in HD versus control brains after adjusting for multiple comparisons (FDR *q-*value < 0.05, see Table 2; see Additional file 3: Table S3 for sequencing read statistics). In HD, 46 miRNAs were identified as significantly up-regulated and 29 as down-regulated in their expression. Hox-related miRNAs had the most extreme, positive fold changes, where miR-10b-5p was 3.9 log2 fold increased, miR-196a-5p was 2.4 log2 fold increased, miR-615-3p was 1.6 log2 fold increased, miR-10b-3p was 1.5 log2 fold increased, and miR-196b-5p was 1.3 log2 fold increased (see Figure 1, see Table 2). Both the 5′ and 3′ mature miRNAs were differentially expressed for eight miRNA precursors (miR-10b, miR-129, miR-1298, miR-142, miR-144, miR-148a, miR-302a, and miR-486). In HD and controls, most 5′-3′ miRNA pairs were positively correlated in their expression, with the exception of miR-1298 in HD and miR-10b and miR-302a in controls (Additional file 4: Table S4).

**Table 2:** **Differentially expressed miRNAs in Huntington’s disease prefrontal cortex**

**miRNA**	**Average expression**	**Original study, N=21**			**Replication study, N=41**			**Combined study, N=64**		
		**logFC**	**p-value**	**FDR q-value**	**logFC**	**p-value**	**FDR q-value**	**logFC**	**p-value**	**FDR q-value**
miR-10b-5p	11.62	4.31	4.56E-11	4.28E-08	3.40	4.30E-12	1.35E-09	3.94	1.28E-20	1.20E-17
miR-196a-5p	2.41	2.18	1.66E-09	7.80E-07	2.13	3.42E-12	1.35E-09	2.35	2.97E-20	1.39E-17
miR-615-3p	1.95	1.28	1.69E-06	3.97E-04	1.73	2.56E-13	2.40E-10	1.59	2.33E-16	7.28E-14
miR-10b-3p	2.02	1.37	4.64E-07	1.45E-04	1.15	2.93E-06	6.88E-04	1.45	2.13E-12	4.98E-10
miR-1298-3p	7.05	−0.56	1.72E-03	9.47E-02	−0.80	1.09E-05	2.04E-03	−0.78	5.52E-09	1.03E-06
miR-196b-5p	2.56	1.05	7.62E-05	1.02E-02	1.06	9.34E-04	5.84E-02	1.31	2.33E-08	3.64E-06
miR-302a-3p	2.28	0.64	6.22E-03	1.94E-01	0.84	3.57E-04	2.79E-02	0.81	3.72E-06	4.98E-04
miR-1247-5p	6.18	0.90	2.05E-05	3.84E-03	0.46	7.81E-03	1.63E-01	0.62	8.47E-06	9.55E-04
miR-144-3p	10.26	0.80	4.48E-02	3.73E-01	1.09	2.63E-04	2.47E-02	1.08	9.16E-06	9.55E-04
miR-223-3p	8.46	0.49	3.95E-02	3.54E-01	0.94	6.20E-05	7.33E-03	0.75	1.94E-05	1.82E-03
miR-3200-3p	9.75	−0.25	8.48E-02	4.65E-01	−0.29	4.20E-03	1.31E-01	−0.32	4.85E-05	4.14E-03
miR-302a-5p	2.99	0.52	2.86E-03	1.28E-01	0.62	1.97E-02	2.66E-01	0.70	5.70E-05	4.46E-03
miR-1264	5.00	−0.24	1.09E-01	5.15E-01	−0.69	3.87E-04	2.79E-02	−0.53	9.49E-05	6.36E-03
miR-6734-5p	2.79	−0.34	1.89E-01	6.19E-01	−1.16	1.63E-05	2.55E-03	−0.79	8.86E-05	6.36E-03
miR-144-5p	9.30	0.51	1.43E-01	5.71E-01	1.13	3.31E-04	2.79E-02	0.94	1.04E-04	6.53E-03
miR-138-2-3p	6.08	−0.44	3.59E-03	1.41E-01	−0.29	3.24E-02	3.01E-01	−0.38	1.43E-04	8.38E-03
miR-431-5p	5.65	−0.49	2.33E-02	3.09E-01	−0.51	7.64E-03	1.63E-01	−0.57	1.60E-04	8.84E-03
miR-132-3p	12.93	−0.48	1.57E-02	2.60E-01	−0.43	2.72E-02	2.89E-01	−0.54	1.99E-04	9.31E-03
miR-200c-3p	3.84	0.46	3.97E-02	3.54E-01	0.26	1.12E-01	4.84E-01	0.48	1.97E-04	9.31E-03
miR-23b-5p	3.18	−0.30	8.92E-02	4.66E-01	−0.62	2.02E-03	9.46E-02	−0.55	1.81E-04	9.31E-03
miR-448	4.02	−0.14	5.66E-01	8.91E-01	−0.80	9.98E-05	1.04E-02	−0.64	2.23E-04	9.96E-03
miR-486-3p	4.84	0.54	7.85E-02	4.57E-01	0.79	4.16E-03	1.31E-01	0.78	2.76E-04	1.04E-02
miR-490-5p	5.56	−0.45	6.53E-02	4.34E-01	−0.56	1.15E-02	2.12E-01	−0.62	2.62E-04	1.04E-02
miR-5695	3.30	0.38	3.04E-02	3.28E-01	0.48	4.02E-03	1.31E-01	0.47	2.73E-04	1.04E-02
miR-885-5p	10.46	−0.31	5.23E-02	4.07E-01	−0.27	3.12E-02	3.01E-01	−0.35	2.77E-04	1.04E-02
miR-1224-5p	8.08	−0.39	3.89E-02	3.54E-01	−0.53	4.83E-03	1.39E-01	−0.49	3.83E-04	1.20E-02
miR-1298-5p	6.43	−0.80	9.80E-03	2.17E-01	−0.65	3.24E-02	3.01E-01	−0.81	3.84E-04	1.20E-02
miR-142-3p	8.13	0.20	3.09E-01	7.41E-01	0.62	1.70E-03	8.39E-02	0.52	3.84E-04	1.20E-02
miR-346	8.21	−0.31	6.05E-02	4.26E-01	−0.27	1.74E-02	2.66E-01	−0.32	3.71E-04	1.20E-02
miR-891a-5p	5.84	0.79	5.16E-05	8.07E-03	0.18	3.68E-01	7.04E-01	0.50	3.69E-04	1.20E-02
miR-16-2-3p	7.23	0.30	3.45E-01	7.53E-01	0.83	6.97E-04	4.67E-02	0.71	3.98E-04	1.21E-02
miR-363-3p	11.07	0.39	1.08E-02	2.20E-01	0.30	2.71E-02	2.89E-01	0.34	4.14E-04	1.21E-02
miR-148a-3p	13.01	0.69	1.70E-02	2.60E-01	0.47	7.34E-02	4.18E-01	0.69	4.57E-04	1.29E-02
miR-199a-5p	7.66	0.69	3.46E-02	3.35E-01	0.66	4.30E-02	3.45E-01	0.82	4.66E-04	1.29E-02
miR-4449	3.25	−0.96	1.83E-03	9.51E-02	−0.86	5.21E-02	3.68E-01	−1.09	5.28E-04	1.42E-02
miR-106a-5p	6.28	0.52	9.97E-03	2.17E-01	0.40	3.15E-02	3.01E-01	0.44	5.64E-04	1.43E-02
miR-142-5p	11.47	0.20	4.43E-01	8.36E-01	0.71	1.66E-03	8.39E-02	0.60	5.77E-04	1.43E-02
miR-549a	3.25	0.57	9.95E-02	4.89E-01	0.86	1.84E-02	2.66E-01	0.95	5.67E-04	1.43E-02
miR-214-5p	3.99	0.81	8.21E-03	2.12E-01	0.42	2.23E-01	5.96E-01	0.84	6.62E-04	1.59E-02
miR-141-3p	5.43	0.48	1.12E-01	5.20E-01	0.34	2.00E-02	2.66E-01	0.47	8.05E-04	1.89E-02
miR-5680	5.39	−0.20	1.92E-01	6.23E-01	−0.41	5.42E-03	1.40E-01	−0.35	9.93E-04	2.27E-02
miR-3065-5p	6.04	0.37	1.10E-01	5.15E-01	0.40	6.86E-03	1.57E-01	0.42	1.04E-03	2.33E-02
miR-224-5p	4.95	0.71	5.90E-02	4.20E-01	0.82	1.98E-02	2.66E-01	0.88	1.19E-03	2.60E-02
miR-4787-3p	5.94	−0.25	1.84E-01	6.15E-01	−0.30	1.29E-02	2.23E-01	−0.33	1.23E-03	2.62E-02
miR-452-5p	4.76	0.32	2.06E-01	6.41E-01	0.68	2.02E-02	2.66E-01	0.67	1.29E-03	2.69E-02
miR-129-1-3p	9.79	−0.42	3.13E-02	3.33E-01	−0.28	5.47E-02	3.79E-01	−0.38	1.36E-03	2.76E-02
miR-4443	5.69	0.92	1.10E-02	2.20E-01	0.41	1.54E-01	5.39E-01	0.75	1.39E-03	2.77E-02
miR-101-5p	9.55	0.30	2.49E-02	3.11E-01	0.20	1.08E-01	4.74E-01	0.28	1.47E-03	2.88E-02
miR-483-5p	4.39	1.03	5.31E-02	4.07E-01	0.78	8.24E-02	4.31E-01	1.16	1.52E-03	2.91E-02
miR-2114-5p	3.41	0.39	3.34E-02	3.33E-01	0.29	1.85E-01	5.72E-01	0.48	1.65E-03	3.09E-02
miR-1185-1-3p	5.32	−0.24	2.34E-01	6.71E-01	−0.43	8.49E-03	1.67E-01	−0.41	1.70E-03	3.12E-02
miR-670-3p	6.70	−0.46	5.50E-02	4.13E-01	−0.39	7.24E-02	4.18E-01	−0.52	1.77E-03	3.19E-02
miR-129-5p	12.39	−0.13	3.31E-01	7.47E-01	−0.50	3.31E-03	1.20E-01	−0.35	1.95E-03	3.22E-02
miR-135b-5p	4.45	−0.49	1.70E-02	2.60E-01	−0.44	5.58E-02	3.82E-01	−0.52	1.97E-03	3.22E-02
miR-194-5p	8.77	0.23	8.25E-02	4.64E-01	0.32	3.68E-02	3.29E-01	0.33	1.99E-03	3.22E-02
miR-208b-3p	6.41	0.46	1.10E-02	2.20E-01	0.28	7.05E-02	4.18E-01	0.36	1.89E-03	3.22E-02
miR-4488	2.97	−1.38	3.79E-04	3.24E-02	−0.91	1.35E-01	5.16E-01	−1.32	1.96E-03	3.22E-02
miR-888-5p	2.83	0.56	3.39E-02	3.35E-01	0.39	7.20E-02	4.18E-01	0.56	1.91E-03	3.22E-02
miR-126-5p	15.88	0.41	2.59E-02	3.16E-01	0.23	6.10E-02	4.03E-01	0.29	2.46E-03	3.88E-02
miR-34c-5p	9.25	−1.09	6.77E-04	4.75E-02	−0.40	1.41E-01	5.26E-01	−0.64	2.48E-03	3.88E-02
miR-218-1-3p	6.08	0.30	5.80E-02	4.20E-01	0.39	2.29E-02	2.76E-01	0.35	2.53E-03	3.89E-02
miR-150-5p	10.20	0.42	2.03E-02	2.84E-01	0.33	6.04E-02	4.02E-01	0.39	2.74E-03	4.11E-02
miR-486-5p	14.08	0.70	7.24E-02	4.52E-01	0.66	4.08E-02	3.39E-01	0.75	2.76E-03	4.11E-02
miR-433-3p	10.55	−0.01	9.48E-01	9.91E-01	−0.36	1.23E-03	7.24E-02	−0.24	2.85E-03	4.18E-02
miR-219b-3p	3.11	−0.46	1.89E-02	2.78E-01	−0.24	3.09E-01	6.46E-01	−0.47	3.05E-03	4.40E-02
miR-548n	2.82	0.09	6.44E-01	9.27E-01	0.64	6.41E-03	1.50E-01	0.52	3.14E-03	4.46E-02
miR-663b	2.20	−0.73	1.49E-02	2.59E-01	−0.59	9.97E-02	4.58E-01	−0.81	3.21E-03	4.50E-02
miR-148a-5p	6.67	0.46	4.67E-02	3.81E-01	0.44	7.58E-02	4.18E-01	0.52	3.31E-03	4.57E-02
miR-29a-3p	15.37	0.20	1.33E-01	5.56E-01	0.22	4.17E-02	3.40E-01	0.23	3.46E-03	4.70E-02
miR-320b	5.63	1.13	1.69E-02	2.60E-01	0.56	1.93E-01	5.78E-01	0.97	3.54E-03	4.74E-02
miR-181a-3p	12.15	−0.43	2.97E-02	3.26E-01	−0.29	9.44E-02	4.51E-01	−0.38	3.60E-03	4.75E-02
miR-153-5p	7.32	0.55	7.08E-03	2.05E-01	0.22	1.80E-01	5.72E-01	0.37	3.78E-03	4.79E-02
miR-28-5p	10.13	0.24	1.37E-01	5.65E-01	0.22	6.84E-02	4.14E-01	0.27	3.75E-03	4.79E-02
miR-7-2-3p	6.06	0.25	8.90E-02	4.66E-01	0.26	4.67E-02	3.59E-01	0.27	3.78E-03	4.79E-02
miR-877-5p	7.14	−0.28	1.28E-01	5.50E-01	−0.29	2.07E-02	2.66E-01	−0.28	3.88E-03	4.85E-02
miR-3687	4.14	−1.37	3.47E-04	3.24E-02	−0.77	1.96E-01	5.78E-01	−1.20	4.25E-03	5.17E-02
miR-4516	3.82	−1.29	3.03E-04	3.16E-02	−0.77	1.72E-01	5.61E-01	−1.13	4.42E-03	5.24E-02
miR-3139	3.01	−0.82	4.64E-04	3.63E-02	0.00	9.86E-01	9.94E-01	−0.48	9.64E-03	8.37E-02
miR-663a	2.56	−1.02	1.61E-04	1.89E-02	−0.46	3.60E-01	6.98E-01	−0.86	1.40E-02	1.04E-01
miR-34b-3p	5.11	−0.92	7.09E-04	4.75E-02	−0.24	3.87E-01	7.16E-01	−0.49	1.77E-02	1.20E-01
miR-1538	2.63	0.20	3.04E-01	7.41E-01	−0.70	6.26E-05	7.33E-03	−0.21	1.30E-01	3.68E-01

To confirm our previously published findings, we re-analyzed the twelve HD and nine controls samples from our original study using our updated sequence analysis pipeline (see Methods) and then used the newly sequenced samples, consisting of 14 HD and 27 control brains, as a replication set. Fourteen miRNAs were significantly differentially expressed (FDR *q*-value < 0.05) in the original set using the updated analysis pipeline, compared to five differentially expressed miRNA in the original study. Fourteen differentially expressed miRNAs were significantly differentially expressed in the replication set and thirteen of these fourteen were significant in the combined sequence analysis. As previously reported, Hox-related miRNA, including miR-10b-5p, were among the most strongly differentially expressed across all three studies (see Table 2).

Firefly Bioworks microRNA assay, a multiplexed, particle-based technology using flow cytometry to measure miRNA levels, was used to quantify and orthogonally validate miRNA differential expression from sequencing (see Methods). A subset of 21 controls and 15 HD samples from the sequencing study were selected for the assay. Sixteen miRNAs with moderately high expression levels were selected for testing and an additional six miRNAs were used as input normalizers (Additional file 5: Table S5). miR-10b-5p was confirmed as significant after correcting for multiple corrections (p-value = 3.0e-10, q-value = 6.6e-9). Seven out of sixteen miRNAs assayed approached but did not reach significance after adjustment in this subset (unadjusted *p*-value < 0.05, miR-10b-5p, miR-194-5p, miR-223-3p, miR-132-3p, miR-144-5p, miR-148a-3p, miR-486-5p). Eight of the remaining nine miRNAs that failed to achieve significance had the same direction of effect (Additional file 5 Table S5). These results were consistent with the reduced power available from this subset.

### Nine miRNAs relate to Vonsattel grade

**Figure 2:**
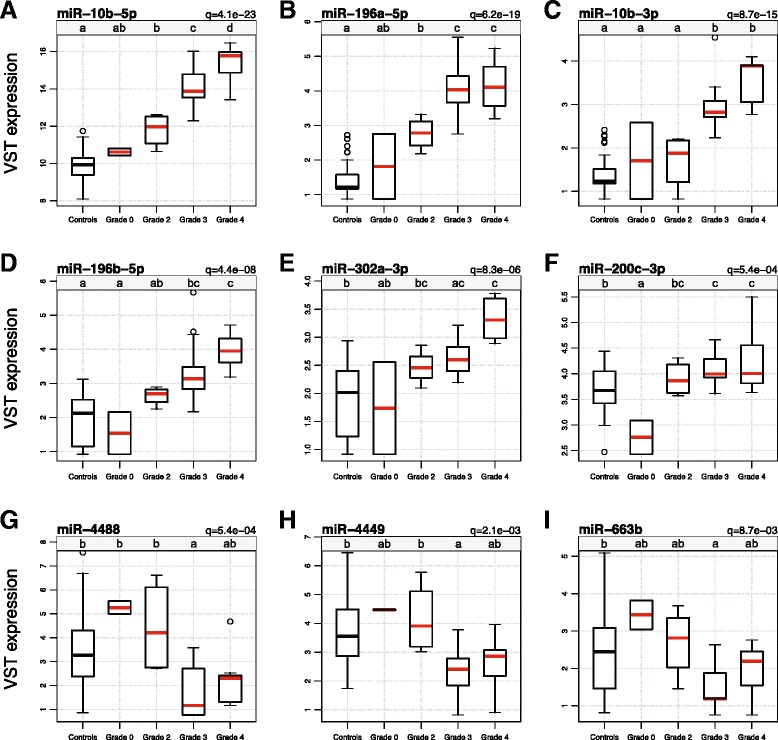
**Nine miRNAs are associated with Vonsattel grade.** In HD brains, expression of differentially expressed miRNA was compared across Vonsattel grades 0–4. Boxplots represent nine FDR-significant miRNAs (**A**. miR-10b-5p, **B**. miR-196a-5p, **C**. miR-10b-3p, **D**. miR-196b-5p, **E**. miR-302a-3p, **F**. miR-200c-3p, **G**. miR-4488, **H**. miR-4449, **I**. miR-663b) (FDR *q* < 0.05, adjusted for 75 contrasts) associated with Vonsattel grade by analysis of variance (ANOVA). X-axes represent Vonsattel grade, classified 0–4 in order of the severity of striatal involvement and Y-axes show the VST expression values after batch correction. Significant differences across grades and controls are denoted by letters in the grey banner above the boxplot, labeled a-d. Groups with different letters are significantly different from one another while those with the same letter are not, after correcting for multiple comparisons. For example, group “a” would be significantly different from group “b” and “c.” Conditions represented by multiple letters indicate no significant difference among those groups. For example, group “ab” would not be significantly different than groups “a” and “b,” but would be different group “c.”

To explore the relationship of miRNA expression to principal clinical aspects of the disease, we next modeled the expression of the 75 differentially expressed miRNAs to the Vonsattel grade of neuropathological involvement. Analysis of variance (ANOVA) was performed to compare the expression of the 75 differentially expressed miRNAs across Vonsattel grade in all 28 (Grade 0–4) HD gene-positive and control brains. 65 miRNA were found to be significant in the ANOVA (FDR-adjusted q-value < 0.05), indicating differential expression may be driven by the difference of controls to specific grades. Next, ANOVA was performed exclusively in HD brains to find whether miRNA differences exist across Vonsattel grades. Nine miRNAs were significant in both ANOVA tests after adjusting for multiple comparisons, indicating a significant difference in the expression of these miRNAs across Vonsattel grades (both FDR *q-*values < 0.05; Additional file 6: Table S6). Last, pairwise comparisons of each grade with the control group were performed using post-hoc Tukey’s HSD (honestly significant different) tests to find specific groups that significantly differed from one another. Figure 2 highlights the nine miRNAs that are associated with grade in order of statistical significance from the ANOVA inclusive of control brains in the test. In Figure 2, significant differences across grade and control groups as determined by Tukey HSD are denoted by letters (a-d) in the grey banner above each boxplot, whereby groups with different letters are significantly different from one another while those which share letters are not.

Several patterns in the relationship of grade to miRNA expression were observed. First, the expression of miR-10b-5p was significant in nearly all comparisons; pairwise contrasts between all grades as well as with the control group were different except for grade 0, although grade 0 was different than grades 2, 3 and 4 (see Figure 2A). Second, the expression of miRNAs in grade 0 brains was rarely different than controls, with the exception of miR-200c-3p, where its expression in grade 0 brains was significantly lower than both controls and grades 2–4 brains (see Figure 2G). Third, the expression of miRNAs in grade 3 and 4 brains appeared relatively similar to one another, with the exception of miR-10b-5p, as mentioned above, and miR-4488, where grade 3 brains were significantly lower than all other groups (see Figure 2D). Although not significant in the HD-only ANOVA, significant pairwise differences between grade 3 and 4 were observed for miR-1298-5p (Bonferroni *q*-value = 3.6e-2) and miR-615-3p (Bonferroni *q* -value = 2.2e-2).

To assess the sensitivity and specificity of miR-10b-5p for predicting HD, area under the curve (AUC) values were calculated using receiver operating characteristic curves (ROC). When comparing HD to controls to predict HD, the AUC was 99.47% (95% confidence level was 98.46%-100%). In a comparison of asymptomatic HD to HD to predict HD status, the AUC was 98.08% (95% confidence level was 92.75-100%) and comparing asymptomatic HD to controls, the AUC was 84.72% (95% CI: 71.09%-98.36%).

### miRNA expression relates to striatal involvement and age of onset in HD

To further elucidate the meaning of the associations of the miRNAs to HD, we examined the relationship between the 75 differentially expressed miRNAs and other salient features of the disease (age at motor onset, disease duration, age at death, and H-V scores of striatal and cortical involvement). To avoid confounding the analysis of these clinical features by the known, strong relationship between *HTT* CAG repeat size and disease pathology and onset [4,28,31,32], CAG-adjusted residuals were calculated for all continuous clinical traits (see Additional file 7: Figure S1).

**Figure 3:**
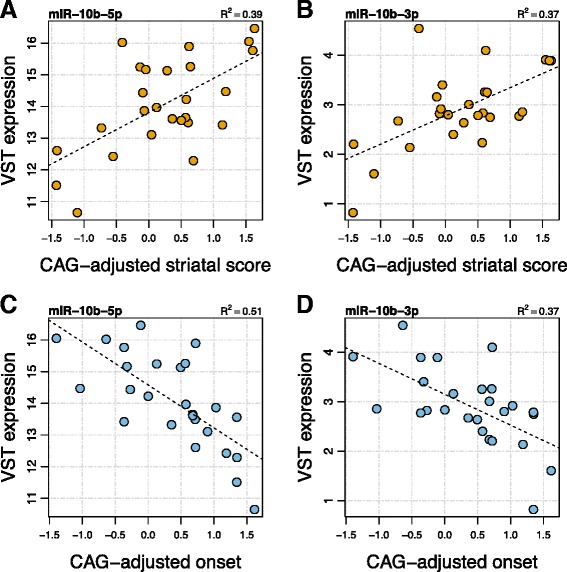
**miR-10b is associated with age of onset and striatal involvement.** In 26 Vonsattel grade 2, 3 and 4 HD brains, both mature miR-10b sequences (−5p and −3p) have FDR-significant relationships to CAG-adjusted Hadzi-Vonsattel striatal score **(A and B)** and CAG-adjusted onset age **(C and D)**. Y-axes show the variance stabilizing transformation expression values after batch correction and shows that miR-10b-5p is expressed at much higher levels than miR-10b-3p. Grade 0 cases are not included, as they have neither onset age nor H-V striatal score.

Using linear regression analysis and applying FDR-adjustment for the 75 comparisons, three miRNAs (miR-10b-5p, miR-10b-3p, miR-302a-3p) were observed to have a significant relationship to CAG-adjusted striatal score (FDR *q-*values = 2.28e-2). All three were significant in the analysis of miRNA expression to Vonsattel grade (see above). Additionally, five miRNAs were identified as having significant association to CAG-adjusted age of onset (miR-10b-5p, FDR *q-*value = 3.49e-3; miR-196a-5p, FDR *q-*value = 1.32e-2; miR-196b-5p, FDR *q-*value = 1.71e-2; miR-10b-3p, FDR *q-*value = 1.71e-2; miR-106a-5p, FDR *q-*value = 1.71e-2). Figure 3 highlights the relationship of miR-10b to CAG-adjusted striatal score and onset, where both 3p and 5p mature sequences of miR-10b were the only miRNA species to have significant, linear association to these two clinical features independent of CAG effect. No FDR-significant relationships of miRNA to disease duration or death age were observed.

No significant relationship of the expression of the 75 differentially expressed miRNA to CAG-adjusted cortical score was observed, although nominal associations were seen. In order to account for the potential impact of cortical involvement on the relationship of miRNA expression to striatal involvement, we performed a multivariate regression analysis modeling miRNA expression to striatal H-V score while correcting for cortical H-V score. After CAG-adjusted cortical score correction, CAG-adjusted striatal score remained significant (miR-10b-5p *p*-value = 0.04, miR-10b-3p *p*-value = 0.01, miR-302a-3p *p*-value = 0.005).

**Figure 4:**
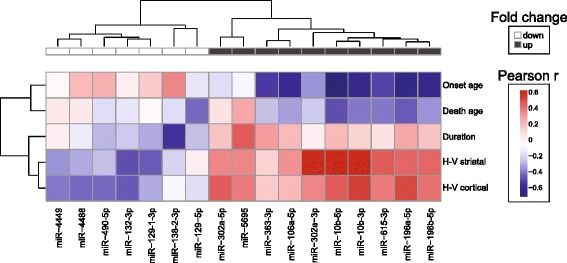
**CAG-adjusted clinical features of HD show patterns of association with miRNA expression.** CAG-adjusted measures of onset age, disease duration, death age, Hadzi-Vonsattel (H-V) striatal and cortical score were correlated with differentially expressed miRNAs in HD brains. miRNAs with at least one nominal *p*-value < 0.05 are shown. Pearson correlation coefficients and features were independently hierarchically clustered. Red boxes indicate positive correlations and blue boxes indicate negative correlations. Seven miRNAs in the left section are down-regulated in HD and the ten miRNAs in the right section are up-regulated. Unsupervised clustering separated miRNA by their direction of fold change.

Last, to characterize the patterns of association of miRNAs to clinical features, Pearson coefficients of the correlation of the expression of the differentially expressed miRNAs to five CAG-adjusted features (onset age, disease duration, death age, striatal score and cortical score) were hierarchically clustered. Grade 0 and controls samples were not included in these analyses. Correlation coefficients rather than beta coefficients were used in order to standardize the direction of effect. Here, we observed differentially expressed miRNAs with correlation *p*-values < 0.05 clustered into distinguishable patterns of association to clinical variables (see Figure 4). Differentially expressed miRNAs increased in HD compared to controls tended to have negative correlations with onset and death, and positive correlations with striatal and cortical score. Conversely, differentially expressed miRNAs with negative relative fold changes had positive correlations with onset and death, and negative correlations with striatal and cortical scores.

### Targets of HD-related miRNAs are associated with nervous system development and transcriptional regulation

To attempt to understand the potential functional impact of miRNA dysregulation in HD, gene ontology enrichment was performed using predicted targets for miRNAs that correlated with clinical features and were suitably annotated in Targetscan (twelve miRNAs in total). 5712 unique mRNA targets for miRNAs with positive fold change in HD (miR-106a/302a-5p, miR-196a/miR-196b, miR-302a-3p, miR-363, miR-10b, miR-615-3p), and 6572 mRNA targets for negative fold change in HD (miR-129-3p, miR-129-5p, miR-132-3p, miR-4449, miR-4488, miR-490-5p) were found using Targetscan [33], and stratified by fold change for gene ontology term (GO) enrichment analysis. Using TopGO’s weight algorithm with Fisher’s Exact Test for gene ontology term enrichment and a weighted p-value cutoff less than *p* < 0.05, 354 GO Biological Processes, 86 GO Molecular Functions and 62 GO Cellular Component terms for mRNA targets of down-regulated miRNA were significant. 260 GO Biological Processes, 78 GO Molecular Functions, 48 GO Cellular Component terms for mRNA targets of up-regulated miRNA were significant.

To make these long lists of GO terms more intelligible, terms were summarized using semantic similarity measures to remove gene-set and GO term redundancy (see Methods).

**Figure 5:**
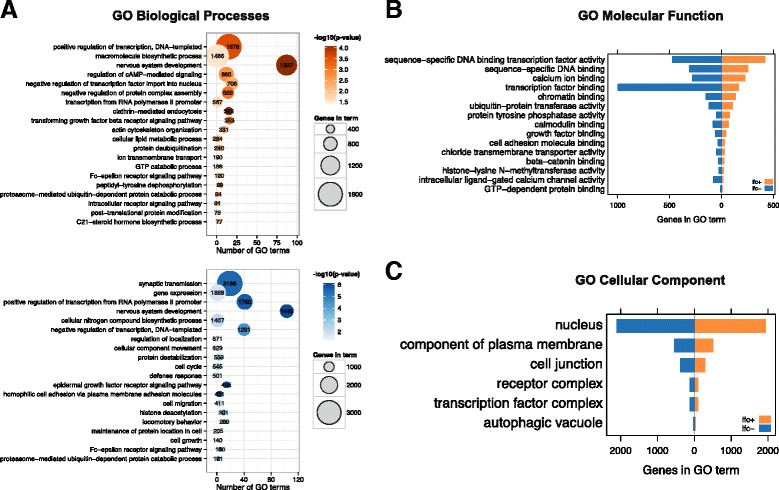
**Gene ontology terms are similar for mRNA targets of clinically relevant de-regulated miRNAs. (A)** Illustrates the overlap in GO Biological Processes between targets of increased miRNA (in orange) and decreased miRNA (in blue) in HD. The x-axis shows the number of gene ontology terms that fall within a given semantic term set, and the y-axis lists the top twenty enriched terms for each set of miRNA targets. Darker colored points represent terms with higher significance and the size of the points represents the union of all genes that fall within a given the term. A number of terms, including “nervous system development” as well as terms relating to transcriptional regulation are shared across up- and down-regulated miRNA target groups. The similarity targets of up-regulated miRNA (in orange) and down-regulated miRNA (in blue) for GO Molecular Function are seen in **(B)** and for GO Cellular Component in **(C)**.

Targets of up- and down-regulated miRNAs had substantial overlap in their overall function. Three of the top twenty collapsed GO Biological Processes terms were shared between the two sets of targets (see Figure 5A). These terms were “nervous system development,” “Fc-epsilon receptor signaling pathway,” and “proteasome − mediated ubiquitin − dependent protein catabolic process.” “Nervous system development” was the most significant term in both sets (Up p = 8.5e-5, Down p = 9.9e-7). The top enriched term was “positive regulation of transcription, DNA-templated”, (N = 1678, p = 2.7e-4) for the positive gene set and “synaptic transmission”, (N = 3166, p = 3.4e-6) for the negative gene set. Of the 78 up-regulated Molecular Function terms and 86 down-regulated terms, fifteen terms were the same (see Figure 5B). Top terms were included “sequence-specific DNA binding transcription factory activity”, “sequence-specific DNA binding” and “calcium ion binding”. Though shared between the two groups, “transcription factor binding” was enriched higher in down-regulated miRNAs than positive ones. For GO Cellular Component, six terms were the same between the two gene sets. These terms included “nucleus” and “cytoplasm” as well as “cell junction” (see Figure 5C).

## Discussion

In a next-generation sequence analysis of small non-coding RNAs in 26 HD and 36 control brains we detected 938 miRNAs and 75 of these were differentially expressed. All five miRNAs reported as differentially expressed in our previous study (miR-10b-5p, miR-196a-5p, miR-196b-5p, miR-615-3p and miR-1247-5p) were significantly differentially expressed in this study [13]. These results were independently validated in the 41 (14 HD and 27 control brains) newly studied brains (see Table 2), and support the presence and robust up-regulation of Hox-related miRNAs in HD brain [13]. The increased number of differentially expressed miRNAs is likely due to an increase in sample size. Increasing our sample size (from N = 21 to N = 62) enhanced the statistical power to detect additional miRNAs with smaller but significant changes in miRNA expression. We believe these miRNA signals are not attributed to a change in BA9 architecture, influenced by neuronal cell death or reactive glial response, because cell numbers between HD and controls from the same brain samples were indistinguishable [13,30].

Dysregulation of several miRNAs from our study have been observed in HD in other contexts. Concordant with our findings, miR-132-3p down-regulation in human HD parietal cortical tissue [15] and in brains of R6/2 and YAC128 HD mouse models has been observed [15,18]. miR-132 is highly enriched in the brain [34,35] and its expression has been shown to affect neuron morphogenesis and enhance neurite outgrowth by suppressing the GTPase-activating protein p250GAP (*p250GAP*/*RICS*) [36]. Another target of miR-132 is acetylcholinesterase (*ACHE*), which encodes an enzyme responsible for the breakdown of the neurotransmitter acetylcholine at the neural synapse [37]. Acetylcholinesterase is critically involved in cognition, and acetylcholinesterase inhibitors are FDA-approved for the treatment of cognitive impairments in Alzheimer’s disease [37]. Thus, decreased miR-132 levels may negatively impact brain health, through the dysregulation of *p250GAP* (limiting its suppression) and *ACHE* (indirectly decreasing acetylcholine levels).

Differentially expressed miRNAs may also target *HTT* transcripts as a response to mutant *HTT* to reduce *HTT* transcriptional levels and limit toxicity. miRNAs that target the *HTT* 3′UTR and reduce *HTT* transcript levels in vitro, miR-148a-5p, miR-150-5p and miR-214-5p, were significantly up-regulated in their expression [21,38]. Although miR-196a does not directly target *HTT* [33], increased miR-196a expression was observed in a primate model of HD and its over-expression *in vitro* and in animal transgenic models suppressed mutant *HTT* expression [24]. The miRNAs with the largest effect in our study, miR-10b-5p, putatively targets *HTT* by binding to two 3′UTR sites (both 7mer-1A seed, positions 2742–2748 and 3301–3307) and may reduce expression of *HTT* although it is not clear whether or not this would be neuroprotective [33].

However, miR-10b-5p also targets brain-derived neurotrophic factor (*BDNF*) [39], a growth factor required for the survival and differentiation of striatal neurons [40]. *BDNF* has been extensively studied in HD [41], as normal huntingtin protein is reported to up-regulate *BDNF* levels, while mutant huntingtin impairs BDNF protein abundance which may consequently lead to death of striatal neurons [42]. Because of the potential biological importance of *BDNF*, and the possibility that miR-10b-5p may diminish translation of *BDNF*, over-expression of miR-10b-5p might be harmful to neuronal cells. However, in Hoss et al. [13], we showed that ectopic expression of miR-10b-5p in PC12 cells expressing a mutant huntingtin fragment enhanced cell survival [13], and miR-10b-5p has been observed to facilitate neurodifferentiation [43]. Given its high levels of differential expression, strong relationship to striatal involvement and age at onset, more research into miR-10b-5p is justified to understand its role in the pathogenesis of HD, its potential as a biomarker of disease progression and its potential as a therapeutic target.

The cell type most responsible for miRNA changes cannot be determined from these data. Tissue homogenate was used for sequencing, so the source of miRNA signal is likely both neuronally and non-neuronally derived. To determine the miRNA cellular specificity in the brain, Jovicic et al. [16] measured miRNA expression in cultured neurons, oligodendrocytes, microglia and astrocytes to find miRNAs enriched for each cell type. Based upon this study, miRNAs found to be specifically enriched in neuronal cultures (miR-129-3p, miR-129-5p, miR-132, miR-135b, miR-431, miR-433) were all down-regulated in our study whereas miRNAs enriched in microglial cultures (miR-126-5p, miR-126-3p, miR-141, miR-142-3p, miR-142-5p, miR-150, miR-200c and miR-223) were all were up-regulated. According to these enrichment categories, microglial activation miRNAs do not relate to clinical features of the disease. Conversely, three neuronal-related miRNAs, miR-129-3p/5p and miR-132, were associated with pathological involvement (see Figure 5). Therefore, we hypothesize that differential expression of those miRNAs related to neuron function may also relate to the HD pathology.

The relationship of the expression of miRNAs with Vonsattel grade suggests expression changes may occur early in the disease process (see Figure 2). Many of these miRNA changes appear present ordinal trends with an increase (miR-10b-5p, miR-10b-3p, miR-302a, miR-196a-5p, miR-196b-5p) or decrease (miR-663b, miR-4488, miR-4449) in their expression across grade. In particular, miR-10b-5p was significantly different across all groups, with the exception of the asymptomatic grade 0 brains and we believe this is an issue of statistical power. It is possible that the expression of these miRNAs may relate to *HTT* aggregation or proteasomal degradation, as intranuclear inclusions are observed in pre-symptomatic HD [44] and the density of aggregate formation continues over the course of the disease. Three miRNAs (miR-10b-3p/5p, miR-302a) related to H-V striatal score, independent of the CAG repeat expansion size and for miR-10b-5p, independent of cortical involvement. These results suggest the relationship of miRNA expression to striatal involvement in the disease is independent of cortical involvement, which is a critical finding, because prefrontal cortex was the source of tissue profiled in these studies.

Based on correlation (see Figure 4), up-regulated miRNAs clustered together based on their relationships to clinical features. Generally, these miRNAs had strong, positive associations to striatal and cortical H-V scores, weak positive association with disease duration and strong negative associations to onset and death age. Down-regulated miRNAs clustered together as well but were less defined in their relationships to clinical features. Most down-regulated miRNAs were inversely associated with H-V scores and duration, opposite to up-regulated miRNAs. These patterns suggest that decreasing up-regulated miRNAs and increasing down-regulated miRNA may be beneficial. However, it remains to be determined which altered miRNAs are compensatory and potentially neuroprotective and which are pathological and neurotoxic. Furthermore, it is unknown whether these changes are consequential, revealing important molecular aspects of the disease process, or are simply innocent by-products.

However, using target analysis and GO term enrichment, we observed predicted targets of both up- and down-regulated miRNAs shared many of the same biological processes and overall systems relating to “nervous system development.” Both sets contained several transcriptional regulation related terms (transcriptional regulation, DNA-dependent or RNA polymerase II, chromatin remodeling, post-transcriptional gene regulation, chromatin remodeling, etc.). Both sets of genes contained terms on metabolism, apoptosis, metal-binding and ubiquitin. Disruption to any of these systems may affect neuron health. Overall, these finding imply both up- and down-regulated miRNAs many be part of the same or similar biological pathways.

## Conclusions

Our findings identify many miRNA alterations in HD brain and a large number of these are related to clinical manifestations of the disease, where the signal is independent of the size of the CAG repeat expansion. The study of Grade 0 cases suggests that miR-10b-5p expression changes may occur pre-symptomatically. Up- and down-regulated miRNAs may target genes in similar biological systems, and these genes are involved in transcriptional regulation, neuronal development and other important aspects surrounding neuron function. These miRNAs represent attractive candidates for predicting onset age and overall health of the striatum in HD. Studies pursuing these miRNAs as potential biomarkers for HD are in progress, as miRNAs may be detectable in peripheral fluids [45] and thus have potential to function as accessible biomarkers for disease stage, rate of progression, treatment efficacy and other important clinical characteristics of HD.

## Methods

### Sample information

Frozen brain tissue from prefrontal cortex Brodmann Area 9 (BA9) was obtained from the Harvard Brain and Tissue Resource Center McLean Hospital, Belmont MA, Banner Sun Health Research Institute, Sun City, Arizona [46] and Human Brain and Spinal Fluid Resource Center VA, West Los Angeles Healthcare Center, Los Angeles, CA. 26 Huntington’s disease (HD) samples, 2 asymptomatic HD gene carriers, and 36 neurologically and neuropathologically normal control samples were selected for the study (Additional file 1: Tables S1 and Additional file 2: Table S2). HD subjects had no evidence of other neurological disease based on neuropathological examination. HD samples and controls were not different in postmortem interval (PMI) (*p*-value = 0.69), RNA integrity number (*p*-value = 0.08) or gender (*p*-value = 0.51) but differed in ages at death (HD mean age =59.5, control mean age =68.6; *p*-value = 0.01) (see Table 1). Asymptomatic HD samples did not differ in age at death (mean age =67.5) in comparison to HD or control samples (control *p*-value = 0.92; HD *p*-value = 0.40). Information on CAG genotype, onset age, death age, disease duration, Vonsattel grade, Hadzi-Vonsattel striatal and cortical scores for HD samples can be found in Additional file 2: Table S2.

Total RNA was isolated using QIAzol Lysis Reagent and purified using miRNeasy MinElute Cleanup columns. RNA quality for sequencing was assessed using either Agilent’s BioAnalyzer 2100 system and RNA 6000 Nano Kits to determine RNA Integrity Number or Agilent 2200 TapeStation and Agilent DNA ScreenTape assay RNA Quality Number. For each brain sample, 1 ug of RNA was used to construct sequencing libraries using Illumina’s TruSeq Small RNA Sample Prep Kit, according to the manufacturer’s protocol, and sequenced using 1x51nt single-end reads on Illumina’s HiSeq 2000 system at Tufts University (http://tucf-genomics.tufts.edu/) or the Michigan State sequencing core facility (http://rtsf.natsci.msu.edu/genomics/).

### miRNA sequence analysis

Reads were quality filtered, removing reads below 80% Q20, using FASTX-toolkit *FASTQ quality filter* (version 0.0.13.2, http://hannonlab.cshl.edu/fastx_toolkit/). Adapter sequence (5′-TGGAATTCTCGGGTGCCAAGG-3′) was removed from the 3′ end of all reads using cutadapt 1.2.1 (http://code.google.com/p/cutadapt/) and reads less than 15 nucleotides in length were discarded [47]. Reads were collapsed using FASTX-toolkit *FASTA/Q collapser*. Reads were aligned to the UCSC human reference genome (build hg19) using Bowtie version 1.0.0, using no mismatch alignments and a limit of 200 multiple mapping instances [48]. Aligned reads that overlapped with the human miRNA annotation, miRBase version 20, (http://www.mirbase.org/ftp.shtml) were identified using BEDTools *IntersectBed* [49]. Reads longer than 27 bases were removed. miRNA reads were counted if ±4 nucleotides from the mature, annotated 5′ start coordinates. Reads that mapped to multiple locations, represented by a single mature miRNA, were recorded as a single miRNA count. Multi-mapped reads represented by multiple mature miRNA annotations were discarded. Additional file 3: Table S3 for read statistics. R version 3.1.0 and Bioconductor 2.1.4 version were used for differential expression analysis. DESeq2 version 1.40.0 was used for estimation of library size and correction, as well as variance-stabilizing transformation (VST) [50,51]. miRNAs with a mean less than 2 raw read counts across all samples were removed. Batch effect was corrected using ComBat with default options through the Bioconductor package sva 3.10 [52,53]. All samples were included in VST and batch correction. Using 36 controls and 26 HD grades 2–4, differential expression analysis was performed with LIMMA version 3.20.8 [54,55], adjusting for age at death in the model. *Q-*values were FDR-adjusted for 938 comparisons. The unprocessed fastq files, normalized miRNA counts and results from miRNA differential expression analysis have been deposited in NCBI’s Gene Expression Omnibus [56], and are accessible through GEO Series accession number GSE64977 (http://www.ncbi.nlm.nih.gov/geo/query/acc.cgi?acc=GSE64977).

### Firefly miRNA assay

A panel of 16 differentially expressed miRNAs with moderate to high expression (miR-10b-5p, miR-194-5p, miR-223-3p, miR-132-3p, miR-144-5p, miR-148a-3p, miR-486-5p, miR-363-3p, miR-199a-5p, miR-16-2-3p, miR-142-3p, miR-34c-5p, miR-129-5p, miR-433-3p, miR-885-5p, miR-346) and six stably expressed miRNAs in sequencing (miR-9-5p, miR-92a-3p, miR-98-5p, miR-101-3p, miR-151a-3p, miR-338-3p) was used for validation. In a 96-well filter plate, Firefly Multimix (Firefly BioWorks, www.fireflybio.com) was incubated with 25ul Hybridization Buffer and 25ul total RNA at a concentration of 1 ng/ul at 37°C for 60 minutes. After rinsing to removing unbound RNA, 75ul of Labeling Buffer was added to each well, and the plate was incubated for 60 minutes at room temperature. Adapted-modified miRNAs were released from the particles using 90°C water, and PCR amplified using a fluorescently-label primer set. PCR product was hybridized to fresh Firefly Multimix for 30 minutes at 37°C and re-suspended in Run Buffer for readout. Particles were scanned on an EMD Millipore Guava 8HT flow cytometer. Raw output was background subtracted, normalized using the geometric mean of the six normalizer miRNAs and log-transformed. LIMMA version 3.20.8 [54] was used to calculate significance.

### HD feature analysis

For analysis of miRNA expression to Vonsattel grade, Tukey HSD statistics and compact letter display were generated by the multcomp R package [57]. CAG-adjusted age of onset was calculated using the logarithmic model from Djousse et al. 2003 [4]. Hadzi-Vonsattel striatal and cortical scores were measured in 523 HD brain samples as previously described [28]. Samples with greater than 55 repeats or missing CAG information were excluded from analysis, leaving 346 samples. To provide robust residual estimates for the subset of samples included in the sequencing project, H-V striatal score, H-V cortical score, death age and disease duration features were corrected for CAG size by modeling each feature to CAG size within the HD dataset (N = 346) and the residuals from the model were extracted for each sample (Additional file 7: Figure S1) [28]. VST-batch corrected counts were used for all subsequent analyses. CAG-adjusted residuals and miRNA expression relationships were analyzed using linear regressions. Covariates (PMI, RIN, age at death) were not included in linear models, as neither PMI nor RIN were determined to have an effect on the outcome of the results. Age at death could not be included in the analysis due to the relationship of age at death and HD clinical pathology. *Q-*values were FDR-adjusted for 75 differentially expressed miRNA contrasts for linear regressions were reported.

For the cluster analysis in Figure 4, Pearson correlations for miRNA expression to clinical feature were performed and those miRNAs with *p*-values < 0.05, without adjustment for multiple comparisons, were reported. Pearson correlation coefficients were hierarchically clustered using Euclidean distance and unsupervised complete clustering method through the R-package *pheatmap* version 0.7.7.

### Target prediction and gene ontology enrichment

Targetscan, release 6.2 [33] was used to select mRNA targets of miRNAs with at least one relationship to clinical feature. Fourteen miRNAs were available on Targetscan and twelve miRNAs had unique seed sequences. Targets were removed with total context scores ≥ −0.1. miRNAs with positive fold change in HD (miR-106a/302a-5p, miR-196a/miR-196b, miR-302a-3p, miR-363, miR-10b, miR-615-3p), and negative fold change in HD (miR-129-3p, miR-129-5p, miR-132-3p, miR-4449, miR-4488, miR-490-5p) were stratified for gene ontology term (GO) enrichment analysis. GO term enrichment for “biological processes,” “molecular function,” and “cellular component,” was performed using topGO [58] with the “weight01” algorithm and Fisher statistic within the R statistical environment. A weighted Fisher *p*-value < 0.05 threshold was used to select significant GO enrichment. Significant terms were collapsed by semantic similarity using the program REVIGO [59], with p-value included for each term and using the “Small (0.5)” similarity setting. The union of genes from REVIGO “parent” terms was calculated using topGO’s *genes.in.term* function.

### Ethics review

This study was reviewed by the Boston University School of Medicine Institutional Review Board (Protocol H-28974), and was approved as exempt because the study involves only tissue collected post-mortem, and consequently not classified as human subjects.

## Additional files

Additional file 1: Table S1. Sample information for 36 control brains used for miRNA-sequencing. Additional file 2: Table S2. Sample information for 28 Huntington’s disease brains used for miRNA-sequencing. Additional file 3: Table S3. Read statistics for miRNA-sequence analysis. Additional file 4: Table S4. Correlation of differentially expressed miRNA precursor pairs. Additional file 5: Table S5. Summary statistics for Firefly BioWorks assay. Additional file 6: Table S6. Linear regression analysis modeled the relationship of miRNA expression and Vonsattel grade. Additional file 7: Figure S1. Association of clinical features to HD CAG repeat size. CAG-adjusted residuals for onset age, death age, duration, H-V striatal score and H-V cortical score were computed from data derived from 346 HD brain samples with CAG repeat sizes <56 from Hadzi et al. [28]. Red dots represent samples studied in these analyses.

## Abbreviations

ANOVA: Analysis of variance
BA9: Brodmann Area 9
CNS: Central nervous system
DNA: Deoxyribonucleic acid
FDR: False discovery rate
GO: Gene ontology
HD: Huntington’s disease
H-V score: Hadzi-Vonsattel score
PCR: Polymerase chain reaction
miRNAs: Micrornas
PMI: Post-mortem interval
RNA: Ribonucleic acid
