# Supplementary figures and images for: miR-10b-5p expression in Huntington’s disease brain relates to age of onset and the extent of striatal involvement
Source: BMC Med Genomics. 2015 Mar 1;8:10. doi: 10.1186/s12920-015-0083-3 (PMC4349621; doi:10.1186/s12920-015-0083-3)

**A**

Onset age

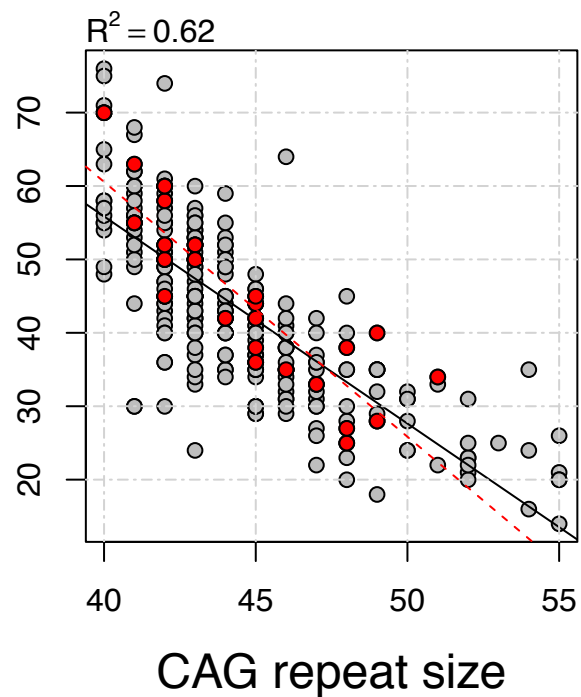**B**

Striatal score

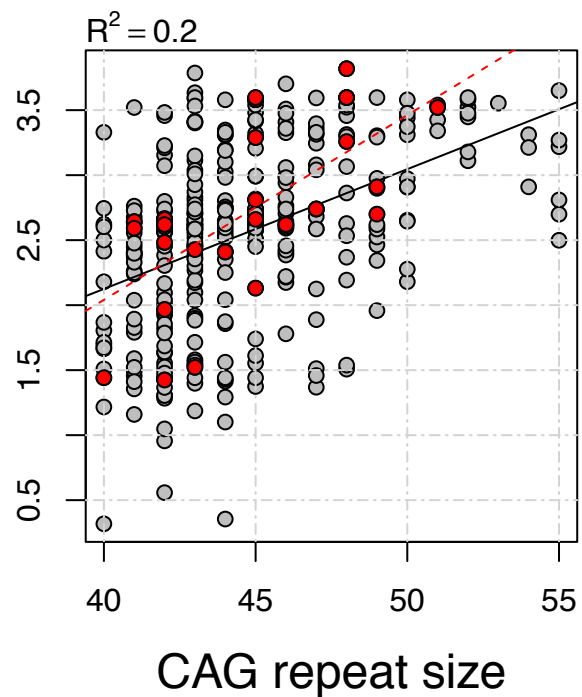**C**

Death age

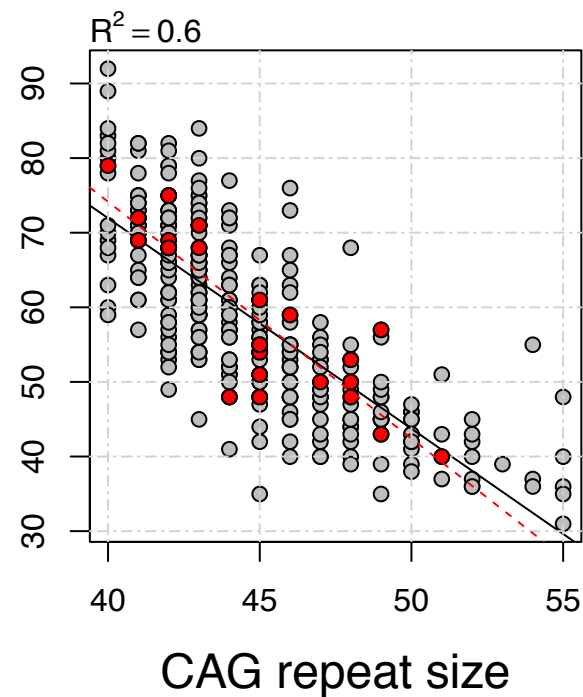**D**

Cortical score

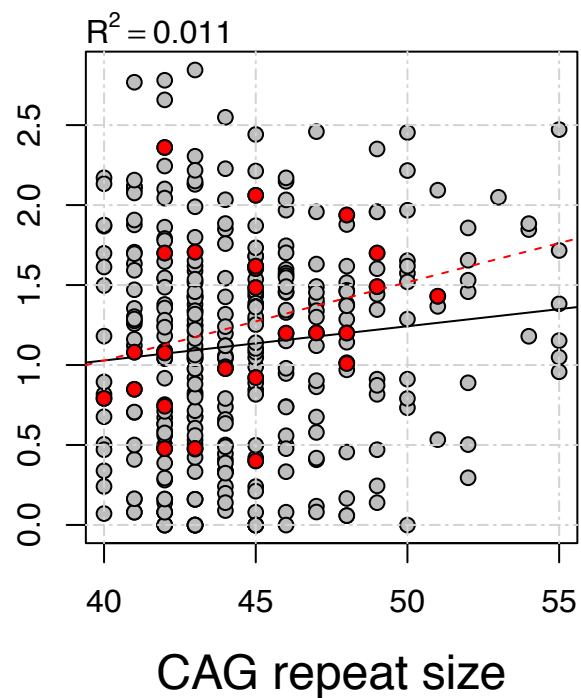**E**

Disease duration

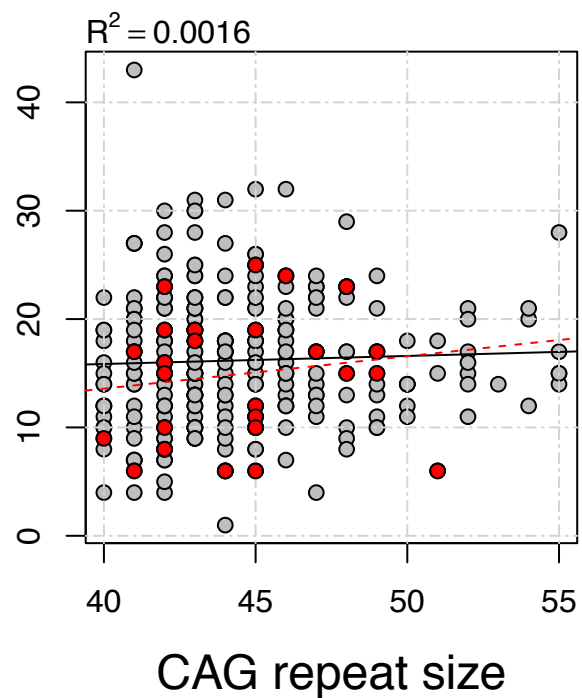

Supplement: Additional file 7: Figure S1. — Association of clinical features to HD CAG repeat size. CAG-adjusted residuals for onset age, death age, duration, H-V striatal score and H-V cortical score were computed from data derived from 346 HD brain samples with CAG repeat sizes <56 from Hadzi et al. [28]. Red dots represent samples studied in these analyses. [file 12920_2015_83_MOESM7_ESM.pdf]
